# Correction: Unified inference of missense variant effects and gene constraints in the human genome

**DOI:** 10.1371/journal.pgen.1009348

**Published:** 2021-01-28

**Authors:** The PLOS Genetics Staff

## Notice of republication

This article was republished on January 14, 2021, to replace an incorrect Fig 4 image included in the article PDF file. The publisher apologizes for the errors. Please download this article again to view the correct version. The originally published, uncorrected article and the republished, corrected articles are provided here for reference.

## Supporting information

S1 File Originally published, uncorrected article. (PDF)

S2 File Republished, corrected article. (PDF)
