# Supplementary material for: Unified inference of missense variant effects and gene constraints in the human genome
Source: PLoS Genet. 2020 Jul 15;16(7):e1008922. doi: 10.1371/journal.pgen.1008922 (PMC7384676; doi:10.1371/journal.pgen.1008922)
Supplement: S1 File — Supplementary figures and tables. (PDF) [file pgen.1008922.s001.pdf]

Supplemental material for:

Unified inference of missense variant effects and gene constraints in the human genome

Yi-Fei Huang

Department of Biology and Huck Institutes of the Life Sciences,
Pennsylvania State University, University Park, PA 16802, USA

Table A: Genomic features for UNEECON.

Feature group	Feature name	Type	Reference	Note
Sequence conservation	SIFT prediction	Binary	[1]	Binary prediction of deleteriousness from SIFT
	LRT prediction	Binary	[1]	Binary prediction of deleteriousness from LRT
	MA prediction	Binary	[1]	Binary prediction of deleteriousness from Mutation Assessor
	PROVEAN prediction	Binary	[1]	Binary prediction of deleteriousness from PROVEAN
	SLR score	Binary	[1]	Raw SLR score
	SIFT score	Numeric	[1]	Raw SIFT score
	LRT omega	Numeric	[1]	Raw LRT score
	MA score	Numeric	[1]	Raw Mutation Assessor score
	PROVEAN score	Numeric	[1]	Raw PROVEAN score
	Grantham score	Numeric	[2]	Raw Grantham score
	HMM entropy	Numeric	[3]	HMM entropy score from SNVBox
	HMM relative entropy	Numeric	[3]	HMM relative entropy score from SNVBox
	dscore	Numeric	[1]	Dscore from PolyPhen-2
	Primate phyloP score	Numeric	[4]	Primate phyloP conservation score
	Mammalian phyloP score	Numeric	[4]	Mammalian phyloP conservation score
	Vertebrate phyloP score	Numeric	[4]	Vertebrate phyloP conservation score
Structural information	PredRSAB	Numeric	[3]	Probability of the residue being buried
	PredRSAI	Numeric	[3]	Probability of the residue being intermediately exposed
	PredRSAE	Numeric	[3]	Probability of the residue being exposed
	PredBFactorF	Numeric	[3]	Probability that the residue's backbone is flexible
	PredBFactorM	Numeric	[3]	Probability that the residue's backbone is intermediately flexible
	PredBFactorS	Numeric	[3]	Probability that the residue's backbone is stiff
	PredStabilityH	Numeric	[3]	Probability that the residue strongly stabilizes folding
	PredStabilityM	Numeric	[3]	Probability that the residue stabilizes folding
	PredStabilityL	Numeric	[3]	Probability that the residue destabilizes folding
	PredSSE	Numeric	[3]	Probability that the secondary structure of the residue is strand
	PredSSH	Numeric	[3]	Probability that the secondary structure of the residue is helix
	PredSSC	Numeric	[3]	Probability that the secondary structure of the residue is loop
Regulatory information	SPIDEX	Numeric	[5]	SPIDEX Splicing score
	Maximum RNA-seq signal	Numeric	[6]	Maximum RNA-seq signal from the Roadmap Epigenomics Project

Table B: Statistical significance of the difference in AUCs between UNEECON and alternative methods in predicting ClinVar missense variants associated with autosomal dominant disorders.

UNEECON	Predicting pathogenic missense variants labeled as "autosomal dominant" in ClinVar	Predicting pathogenic missense variants within autosomal dominant genes
vs MPC	0.04618 *	3.190e-12 **
vs LASSIE	0.0001567 **	0.2367
vs PrimateAI	0.04633 *	8.233e-11 **
vs Eigen	0.001115 **	3.004e-05 **
vs CADD	4.869e-05 **	1.077e-08 **
vs RVIS	3.155e-61 **	1.468e-96 **
vs pLI	5.414e-51 **	5.065e-108 **
vs CCR	2.082e-19 **	1.832e-60 **

The numbers represent p-values from the DeLong test [7]. **: p-value < 0.01; *: p-value < 0.05.

Table C: Statistical significance of the difference in AUCs between UNEECON-G and alternative methods in predicting disease genes and essential genes.

UNEECON-G	HI gene	Autosomal dominant gene	MGI essential gene	CRISPR essential gene
vs pLI	0.4504	5.541e-09**	5.153e-08**	3.893e-19**
vs mis-z	3.514e-8**	2.530e-12**	1.449e-28**	0.0001982**
vs RVIS	1.007e-12**	1.852e-12**	1.969e-25**	0.0002210**
vs GDI	1.930e-08**	4.220e-12**	3.719e-39**	1.043e-26**

The numbers represent p-values from the DeLong test [7]. **: p-value < 0.01; *: p-value < 0.05.

Table D: Enrichment of Reactome pathways in the 956 genes intolerant to both missense and loss-of-function mutations. The 956 genes tolerant to missense but not to loss-of-function mutations are utilized as the background gene set.

Category	Fold enrichment	*p*-value	FDR
Opioid Signalling (R-HSA-111885)	16.95	1.36E-04	1.60E-02
Neurotransmitter receptors and postsynaptic signal transmission (R-HSA-112314)	11.96	2.72E-08	1.04E-05
Transmission across Chemical Synapses (R-HSA-112315)	8.47	3.32E-10	2.54E-07
Neuronal System (R-HSA-112316)	4.20	1.58E-10	2.42E-07
mRNA Splicing (R-HSA-72172)	3.99	2.25E-06	5.75E-04
mRNA Splicing - Major Pathway (R-HSA-72163)	3.99	2.25E-06	4.92E-04
G2/M Transition (R-HSA-69275)	3.29	5.27E-04	4.75E-02
Processing of Capped Intron-Containing Pre-mRNA (R-HSA-72203)	2.83	3.63E-05	5.05E-03
Innate Immune System (R-HSA-168249)	2.21	2.56E-05	3.92E-03
Metabolism of RNA (R-HSA-8953854)	2.12	4.32E-06	8.27E-04
Developmental Biology (R-HSA-1266738)	1.85	7.35E-06	1.25E-03
Axon guidance (R-HSA-422475)	1.85	3.58E-04	3.65E-02
Metabolism of proteins (R-HSA-392499)	1.77	3.51E-07	1.08E-04
Post-translational protein modification (R-HSA-597592)	1.68	1.48E-04	1.61E-02
Metabolism (R-HSA-1430728)	1.64	5.19E-04	4.97E-02
Signal Transduction (R-HSA-162582)	1.46	3.63E-05	4.63E-03
Unclassified (UNCLASSIFIED)	.68	1.63E-09	8.34E-07

Table E: Enrichment of Gene Ontology (molecular function) terms in the 956 genes intolerant to both missense and loss-of-function mutations. The 956 genes tolerant to missense but not to loss-of-function mutations are utilized as the background gene set.

Category	Fold enrichment	*p*-value	FDR
potassium channel activity (GO:0005267)	16.95	1.36E-04	4.29E-03
potassium ion transmembrane transporter activity (GO:0015079)	5.23	8.46E-04	2.04E-02
ligand-gated channel activity (GO:0022834)	4.19	2.34E-03	4.35E-02
ligand-gated ion channel activity (GO:0015276)	4.19	2.34E-03	4.16E-02
GTPase activity (GO:0003924)	3.49	2.09E-04	6.13E-03
ion transmembrane transporter activity (GO:0015075)	3.19	2.81E-05	1.44E-03
cation transmembrane transporter activity (GO:0008324)	3.06	1.23E-04	4.58E-03
inorganic cation transmembrane transporter activity (GO:0022890)	3.06	1.23E-04	4.20E-03
pyrophosphatase activity (GO:0016462)	2.76	1.44E-05	1.47E-03
nucleoside-triphosphatase activity (GO:0017111)	2.76	1.44E-05	1.18E-03
hydrolase activity, acting on acid anhydrides, in phosphorus-containing anhydrides (GO:0016818)	2.76	1.44E-05	9.83E-04
hydrolase activity, acting on acid anhydrides (GO:0016817)	2.76	1.44E-05	8.43E-04
mRNA binding (GO:0003729)	2.68	1.83E-03	3.76E-02
hydrolase activity (GO:0016787)	2.17	1.53E-07	2.09E-05
transmembrane transporter activity (GO:0022857)	2.13	4.26E-04	1.16E-02
transporter activity (GO:0005215)	2.12	5.97E-05	2.45E-03
RNA binding (GO:0003723)	1.94	5.96E-04	1.53E-02
protein kinase activity (GO:0004672)	1.91	9.95E-04	2.27E-02
phosphotransferase activity, alcohol group as acceptor (GO:0016773)	1.87	9.96E-04	2.15E-02
catalytic activity (GO:0003824)	1.81	1.27E-12	5.20E-10
transferase activity, transferring phosphorus-containing groups (GO:0016772)	1.73	2.24E-03	4.37E-02
transferase activity (GO:0016740)	1.73	2.83E-05	1.29E-03
Unclassified (UNCLASSIFIED)	.69	6.78E-11	1.39E-08

Table F: Enrichment of Gene Ontology (biological process) terms in the 956 genes intolerant to both missense and loss-of-function mutations. The 956 genes tolerant to missense but not to loss-of-function mutations are utilized as the background gene set.

Category	Fold enrichment	*p*-value	FDR
regulation of membrane potential (GO:0042391)	6.73	2.96E-05	5.25E-03
RNA splicing (GO:0008380)	5.13	7.19E-06	8.91E-03
mRNA splicing, via spliceosome (GO:0000398)	4.98	1.23E-05	7.62E-03
RNA splicing, via transesterification reactions with bulged adenosine as nucleophile (GO:0000377)	4.98	1.23E-05	5.08E-03
RNA splicing, via transesterification reactions (GO:0000375)	4.98	1.23E-05	3.81E-03
RNA processing (GO:0006396)	3.57	2.56E-05	6.34E-03
trans-synaptic signaling (GO:0099537)	3.41	6.53E-05	9.00E-03
synaptic signaling (GO:0099536)	3.41	6.53E-05	8.10E-03
anterograde trans-synaptic signaling (GO:0098916)	3.32	1.03E-04	1.07E-02
chemical synaptic transmission (GO:0007268)	3.32	1.03E-04	9.87E-03
regulation of biological quality (GO:0065008)	2.28	4.91E-04	4.35E-02
intracellular signal transduction (GO:0035556)	2.20	6.70E-05	7.55E-03
signal transduction (GO:0007165)	1.75	3.00E-05	4.64E-03
cellular response to stimulus (GO:0051716)	1.66	2.64E-05	5.45E-03

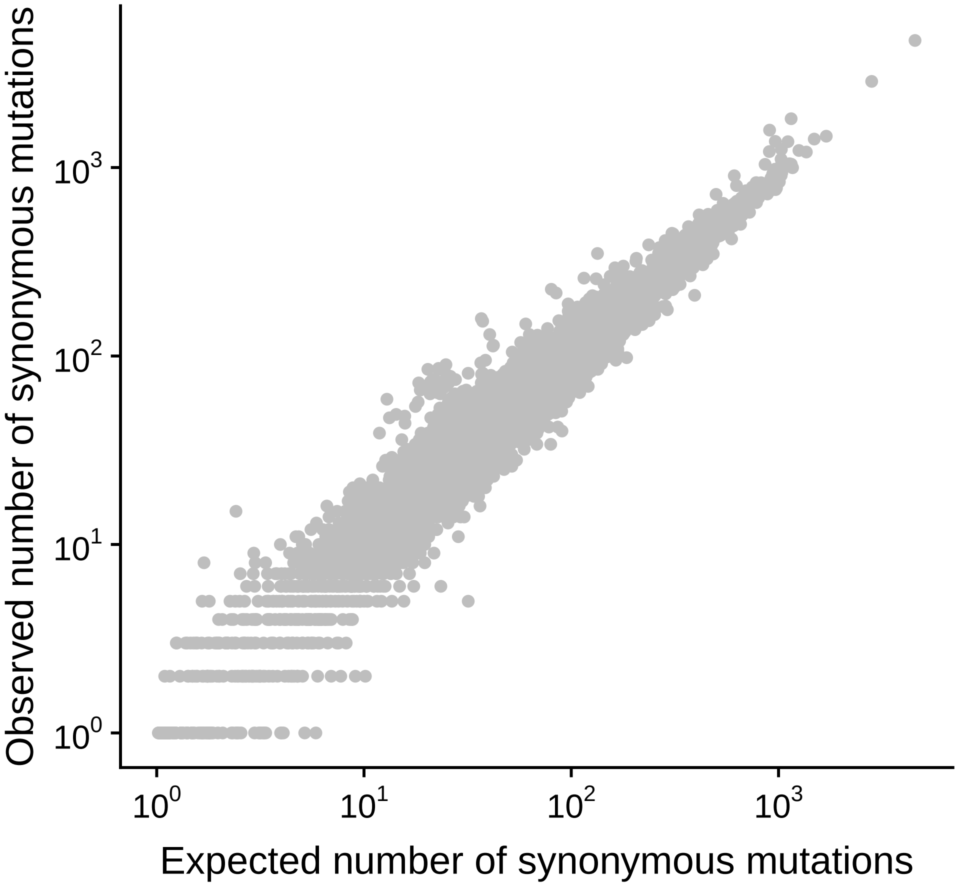

Fig A: Correlation between the expected and the obeserved numbers of synonymous mutations across protein-coding genes under the neutral mutation model. The observed number of synonymous mutations for each gene is derived from the gnomAD exome sequencing data. The expected number of synonymous mutations is predicted by UNEECON's context-dependent mutation model.

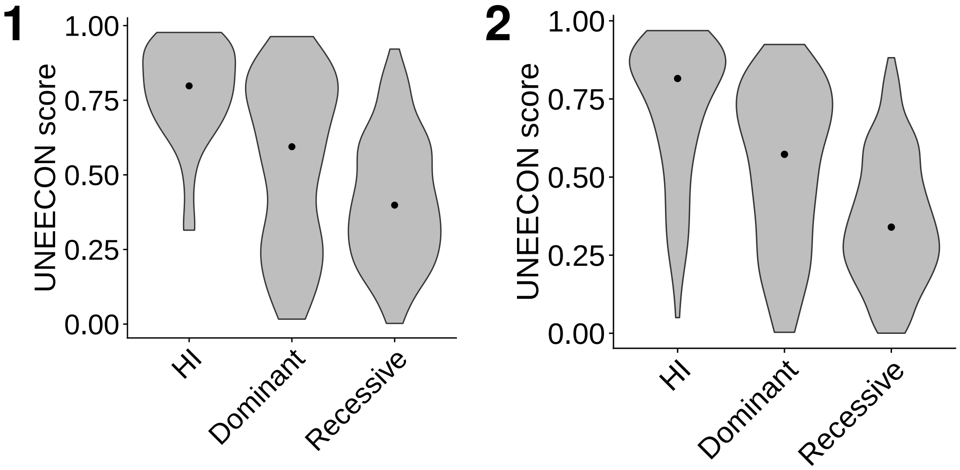

Fig B: Distributions of UNEECON scores in functional protein sites of disease-causing genes. **(1)** Distributions of UNEECON scores estimated for potential missense mutations in enzyme active sites of haploinsufficient (HI) genes [8], autosomal dominant disease genes [9, 10], and autosomal recessive disease genes [9, 10]. **(2)** Distributions of UNEECON scores estimated for potential missense mutations in ligand binding sites of haploinsufficient (HI) genes [8], autosomal dominant disease genes [9, 10], and autosomal recessive disease genes [9, 10]. The black dots indicate the median UNEECON score of each group of functional sites.

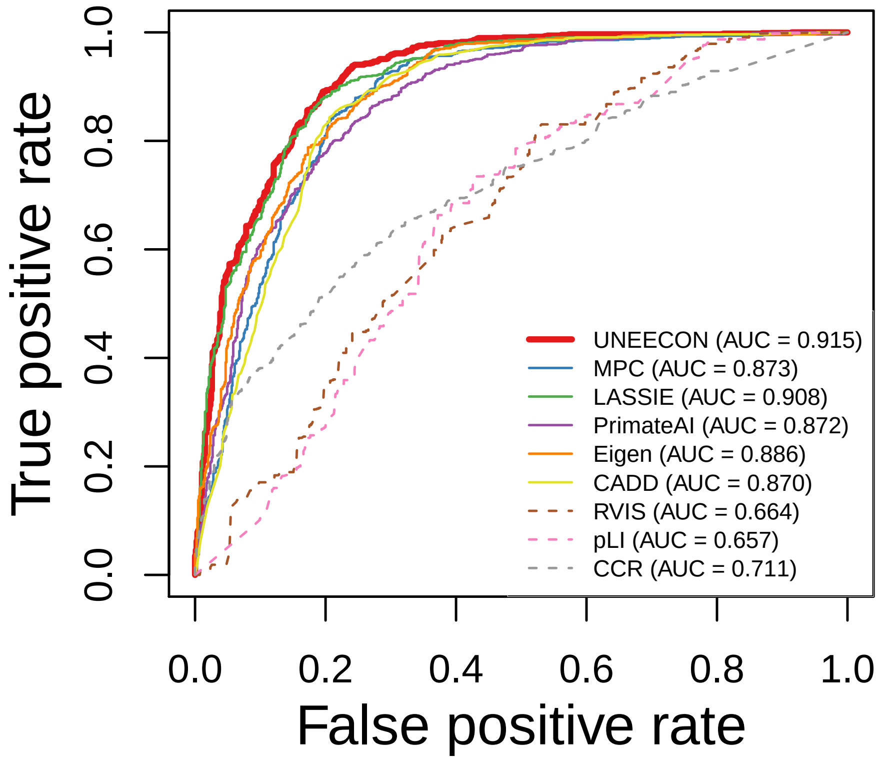

Fig C: Performance of UNEECON and alternative methods in predicting ClinVar pathogenic variants within autosomal dominant genes. Benign missense variants from ClinVar are utilized as negative controls.

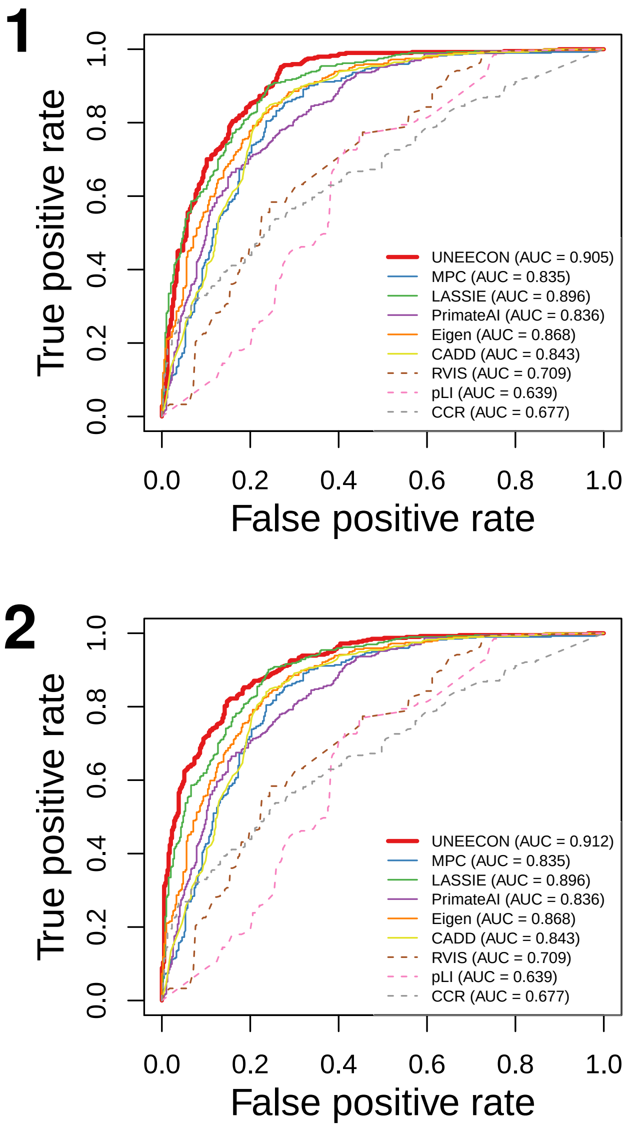

Fig D: Predictive power of various methods for distinguishing pathogenic missense variants from benign missense variants in “mixed” genes. A “mixed” gene is an autosomal dominant disease gene containing at least one pathogenic missense variant and one benign missense variant in ClinVar. **(1)** Comparison of the performance of UNEECON trained on all genes with that of alternative methods. **(2)** Comparison of the performance of UNEECON trained on a dataset without ClinVar disease genes with that of alternative methods.

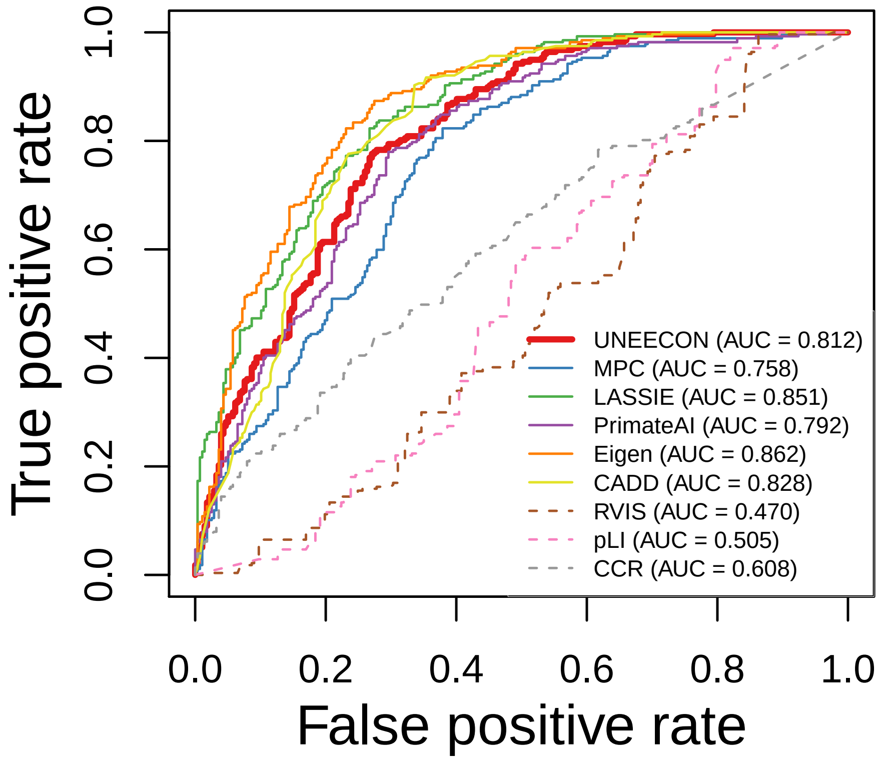

Fig E: Performance of UNEECON and alternative methods in predicting CinVar pathogenic variants with an autosomal recessive mode of inheritance. Benign missense variants from ClinVar are utilized as negative controls.

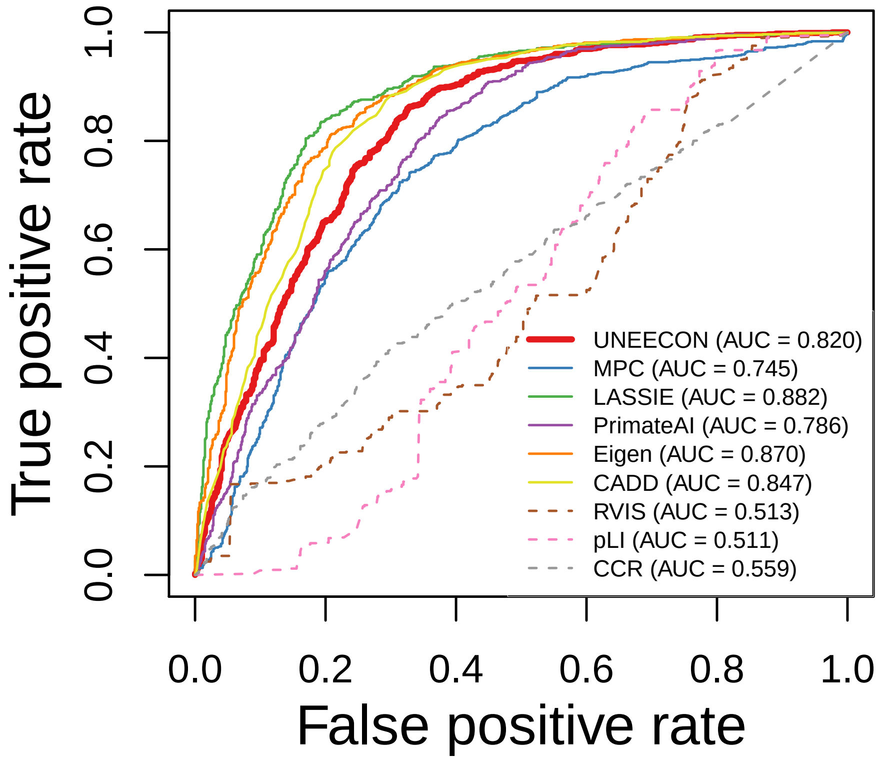

Fig F: Performance of UNEECON and alternative methods in predicting ClinVar pathogenic variants within autosomal recessive disease genes. Benign missense variants from ClinVar are utilized as negative controls.

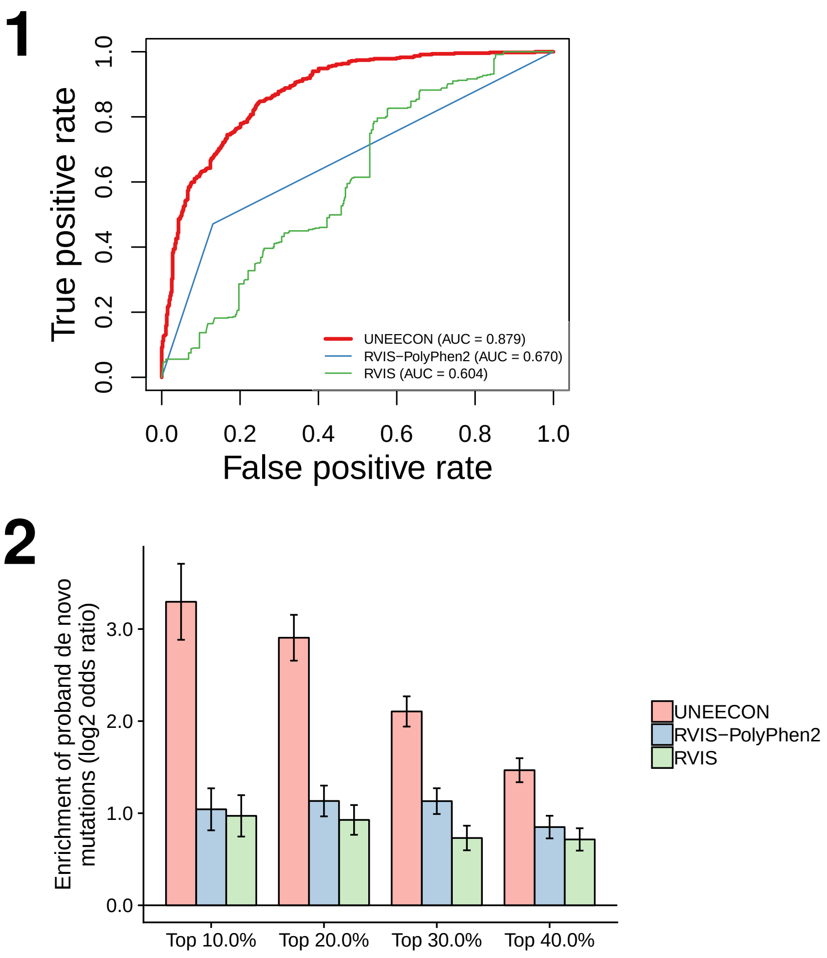

Fig G: Predictive power of UNEECON, RVIS, and a heuristic method combining RVIS and PolyPhen-2 (RVIS-PolyPhen2 [11]) in separating pathogenic missense variants from benign missense variants. **(1)** Performance in predicting autosomal dominant pathogenic variants from ClinVar [12]. True positive and true negative rates correspond to the fractions of pathogenic and benign variants exceeding various thresholds, respectively. AUC corresponds to the area under the receiver operating characteristic curve. **(2)** Enrichment of predicted deleterious *de novo* variants in individuals affected by developmental disorders [13]. The y-axis corresponds to the $\log_2$ odds ratio of the enrichment of predicted deleterious variants in the affected individuals for a given percentile threshold. The x-axis corresponds to the various percentile threshold values used in the enrichment analysis. Error bars represent the standard error of the $\log_2$ odds ratio.

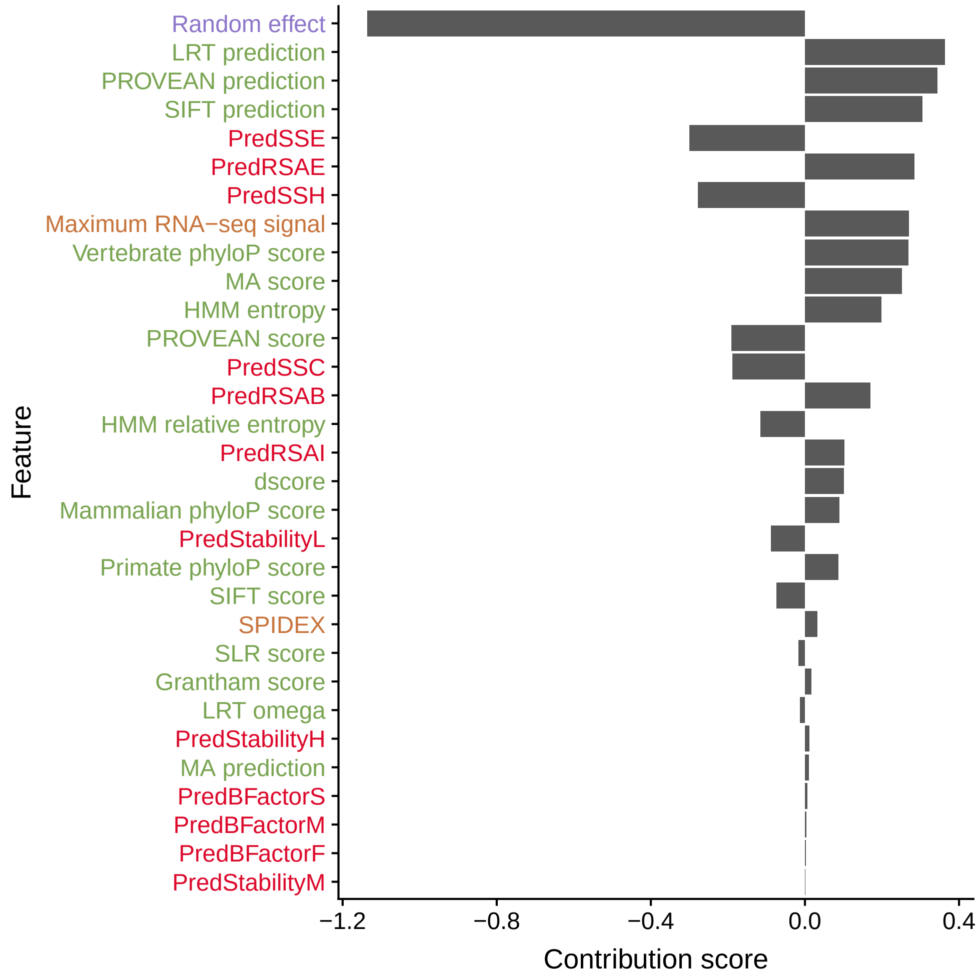

Fig H: Feature contribution scores from the linear UNEECON model. A positive contribution score suggests that the corresponding feature is positively correlated with the strength of selection, while a negative contribution score suggests that the corresponding feature is negatively correlated with the strength of selection. The colors of feature names correspond to four groups: gene-level random effect (purple), sequence conservation (green), structural information (red), and regulatory information (orange).

References

[1] Liu, X., Jian, X., Eric, B.: dbNSFP v2.0: a database of human non-synonymous SNVs and their functional predictions and annotations. Human Mutation **34**(9), 2393–2402 (2013)

[2] Grantham, R.: Amino acid difference formula to help explain protein evolution. Science **185**(4154), 862–864 (1974)

[3] Wong, W.C., Kim, D., Carter, H., Diekhans, M., Ryan, M.C., Karchin, R.: CHASM and SNVBox: toolkit for detecting biologically important single nucleotide mutations in cancer. Bioinformatics **27**(15), 2147–2148 (2011)

[4] Pollard, K.S., Hubisz, M.J., Rosenbloom, K.R., Siepel, A.: Detection of nonneutral substitution rates on mammalian phylogenies. Genome Research **20**(1), 110–121 (2010)

[5] Xiong, H.Y., Alipanahi, B., Lee, L.J., Bretschneider, H., Merico, D., Yuen, R.K.C., Hua, Y., Gueroussov, S., Najafabadi, H.S., Hughes, T.R., Morris, Q., Barash, Y., Krainer, A.R., Jojic, N., Scherer, S.W., Blencowe, B.J., Frey, B.J.: The human splicing code reveals new insights into the genetic determinants of disease. Science **347**, 1254806 (2015)

[6] Roadmap Epigenomics Consortium, Kundaje, A., Meuleman, W., Ernst, J., Bilenky, M., Yen, A., Heravi-Moussavi, A., Kheradpour, P., Zhang, Z., Wang, J., Ziller, M.J., Amin, V., Whitaker, J.W., Schultz, M.D., Ward, L.D., Sarkar, A., Quon, G., Sandstrom, R.S., Eaton, M.L., Wu, Y.-C., Pfenning, A.R., Wang, X., Claussnitzer, M., Liu, Y., Coarfa, C., Harris, R.A., Shoresh, N., Epstein, C.B., Gjoneska, E., Leung, D., Xie, W., Hawkins, R.D., Lister, R., Hong, C., Gascard, P., Mungall, A.J., Moore, R., Chuah, E., Tam, A., Canfield, T.K., Hansen, R.S., Kaul, R., Sabo, P.J., Bansal, M.S., Carles, A., Dixon, J.R., Farh, K.-H., Feizi, S., Karlic, R., Kim, A.-R., Kulkarni, A., Li, D., Lowdon, R., Elliott, G., Mercer, T.R., Neph, S.J., Onuchic, V., Polak, P., Rajagopal, N., Ray, P., Sallari, R.C., Siebenthall, K.T., Sinnott-Armstrong, N.A., Stevens, M., Thurman, R.E., Wu, J., Zhang, B., Zhou, X., Beaudet, A.E., Boyer, L.A., De Jager, P.L., Farnham, P.J., Fisher, S.J., Haussler, D., Jones, S.J.M., Li, W., Marra, M.A., McManus, M.T., Sunyaev, S., Thomson, J.A., Tlsty, T.D., Tsai, L.-H., Wang, W., Waterland, R.A., Zhang, M.Q., Chadwick, L.H., Bernstein, B.E., Costello, J.F., Ecker, J.R., Hirst, M., Meissner, A., Milosavljevic, A., Ren, B., Stamatoyannopoulos, J.A., Wang, T., Kellis, M., Consortium, R.E.: Integrative analysis of 111 reference human epigenomes. Nature **518**(7539), 317–330 (2015)

[7] DeLong, E.R., DeLong, D.M., Clarke-Pearson, D.L.: Comparing the areas under two or more correlated receiver operating characteristic curves: a nonparametric approach. Biometrics **44**, 837–45 (1988)

[8] Rehm, H.L., Berg, J.S., Brooks, L.D., Bustamante, C.D., Evans, J.P., Landrum, M.J., Ledbetter, D.H., Maglott, D.R., Martin, C.L., Nussbaum, R.L., Plon, S.E., Ramos, E.M., Sherry, S.T., Watson, M.S.: ClinGen – the clinical genome resource. New England Journal of Medicine **372**(23), 2235–2242 (2015)

[9] Blekhman, R., Man, O., Herrmann, L., Boyko, A.R., Indap, A., Kosiol, C., Bustamante, C.D., Teshima, K.M., Przeworski, M.: Natural selection on genes that underlie human disease susceptibility. Current Biology **18**(12), 883–889 (2008)

[10] Berg, J.S., Adams, M., Nassar, N., Bizon, C., Lee, K., Schmitt, C.P., Wilhelmsen, K.C., Evans, J.P.: An informatics approach to analyzing the incidentalome. Genetics In Medicine **15**, 36 (2012)

[11] Petrovski, S., Wang, Q., Heinzen, E.L., Allen, A.S., Goldstein, D.B.: Genic intolerance to functional variation and the interpretation of personal genomes. PLOS Genetics **9**(8), 1003709 (2013)

[12] Landrum, M.J., Lee, J.M., Riley, G.R., Jang, W., Rubinstein, W.S., Church, D.M., Maglott, D.R.: ClinVar: public archive of relationships among sequence variation and human phenotype. Nucleic Acids Research **42**(D1), 980–985 (2014)

[13] Deciphering Developmental Disorders Study, McRae, J.F., Clayton, S., Fitzgerald, T.W., Kaplanis, J., Prigmore, E., Rajan, D., Sifrim, A., Aitken, S., Akawi, N., Alvi, M., Ambridge, K., Barrett, D.M., Bayzetinova, T., Jones, P., Jones, W.D., King, D., Krishnappa, N., Mason, L.E., Singh, T., Tivey, A.R., Ahmed, M., Anjum, U., Archer, H., Armstrong, R., Awada, J., Balasubramanian, M., Banka, S., Baralle, D., Barnicoat, A., Batstone, P., Baty, D., Bennett, C., Berg, J., Bernhard, B., Bevan, A.P., Bitner-Glindzicz, M., Blair, E., Blyth, M., Bohanna, D., Bourdon, L., Bourn, D., Bradley, L., Brady, A., Brent, S., Brewer, C., Brunstrom, K., Bunyan, D.J., Burn, J., Canham, N., Castle, B., Chandler, K., Chatzimichali, E., Cilliers, D., Clarke, A., Clasper, S., Clayton-Smith, J., Clowes, V., Coates, A., Cole, T., Colgiu, I., Collins, A., Collinson, M.N., Connell, F., Cooper, N., Cox, H., Cresswell, L., Cross, G., Crow, Y., DAlessandro, M., Dabir, T., Davidson, R., Davies, S., de Vries, D., Dean, J., Deshpande, C., Devlin, G., Dixit, A., Dobbie, A., Donaldson, A., Donnai, D., Donnelly, D., Donnelly, C., Douglas, A., Douzgou, S., Duncan, A., Eason, J., Ellard, S., Ellis, I., Elmslie, F., Evans, K., Everest, S., Fendick, T., Fisher, R., Flinter, F., Foulds, N., Fry, A., Fryer, A., Gardiner, C., Gaunt, L., Ghali, N., Gibbons, R., Gill, H., Goodship, J., Goudie, D., Gray, E., Green, A., Greene, P., Greenhalgh, L., Gribble, S., Harrison, R., Harrison, L., Harrison, V., Hawkins, R., He, L., Hellens, S., Henderson, A., Hewitt, S., Hildyard, L., Hobson, E., Holden, S., Holder, M., Holder, S., Hollingsworth, G., Homfray, T., Humphreys, M., Hurst, J., Hutton, B., Ingram, S., Irving, M., Islam, L., Jackson, A., Jarvis, J., Jenkins, L., Johnson, D., Jones, E., Josifova, D., Joss, S., Kaemba, B., Kazembe, S., Kelsell, R., Kerr, B., Kingston, H., Kini, U., Kinning, E., Kirby, G., Kirk, C., Kivuva, E., Kraus, A., Kumar, D., Kumar, V.K.A., Lachlan, K., Lam, W., Lampe, A., Langman, C., Lees, M., Lim, D., Longman, C., Lowther, G., Lynch, S.A., Magee, A., Maher, E., Male, A., Mansour, S., Marks, K., Martin, K., Maye, U., McCann, E., McConnell, V., McEntagart, M., McGowan, R., McKay, K., McKee, S., McMullan, D.J., McNerlan, S., McWilliam, C., Mehta, S., Metcalfe, K., Middleton, A., Miedzybrodzka, Z., Miles, E., Mohammed, S., Montgomery, T., Moore, D., Morgan, S., Morton, J., Mugalaasi, H., Murday, V., Murphy, H., Naik, S., Nemeth, A., Nevitt, L., Newbury-Ecob, R., Norman, A., OShea, R., Ogilvie, C., Ong, K.-R., Park, S.-M., Parker, M.J., Patel, C., Paterson, J., Payne, S., Perrett, D., Phipps, J., Pilz, D.T., Pollard, M., Pottinger, C., Poulton, J., Pratt, N., Prescott, K., Price, S., Pridham, A., Procter, A., Purnell, H., Quarrell, O., Ragge, N., Rahbari, R., Randall, J., Rankin, J., Raymond, L., Rice, D., Robert, L., Roberts, E., Roberts, J., Roberts, P., Roberts, G., Ross, A., Rosser, E., Saggar, A., Samant, S., Sampson, J., Sandford, R., Sarkar, A., Schweiger, S., Scott, R., Scurr, I., Selby, A., Seller, A., Sequeira, C., Shannon, N., Sharif, S., Shaw-Smith, C., Shearing, E., Shears, D., Sheridan, E., Simonic, I., Singzon, R., Skitt, Z., Smith, A., Smith, K., Smithson, S., Sneddon, L., Splitt, M., Squires, M., Stewart, F., Stewart, H., Straub, V., Suri, M., Sutton, V., Swaminathan, G.J., Sweeney, E., Tatton-Brown, K., Taylor, C., Taylor, R., Tein, M., Temple, I.K., Thomson, J., Tischkowitz, M., Tomkins, S., Torokwa, A., Treacy, B., Turner, C., Turnpenny, P., Tysoe, C., Vandersteen, A., Varghese, V., Vasudevan, P., Vijayarangakannan, P., Vogt, J., Wakeling, E., Wallwark, S., Waters, J., Weber, A., Wellesley, D., Whiteford, M., Widaa, S., Wilcox, S., Wilkinson, E., Williams, D., Williams, N., Wilson, L., Woods, G., Wragg, C., Wright, M., Yates, L., Yau, M., Nellaker,

C., Parker, M., Firth, H.V., Wright, C.F., FitzPatrick, D.R., Barrett, J.C., Hurles, M.E.: Prevalence and architecture of de novo mutations in developmental disorders. Nature **542**, 433–438 (2017)
