# Supplementary material for: Unified inference of missense variant effects and gene constraints in the human genome
Source: PLoS Genet. 2020 Jul 15;16(7):e1008922. doi: 10.1371/journal.pgen.1008922 (PMC7384676; doi:10.1371/journal.pgen.1008922)
Supplement: Attachment — Submitted filename: Response to the reviewers.pdf [file pgen.1008922.s003.pdf]

Response to Reviewers

I thank the reviewers for their careful reading of my manuscript and their thoughtful and constructive suggestions for improvement. My item-by-item responses are interleaved with their comments below (in blue). Note that I used a program, latexdiff, to highlight the changes in my manuscript. Any removed words were crossed out and colored red, whereas added words were colored blue and underlined with a squiggle. When equations were changed, additions were colored blue and removals were colored red.

Reviewer #1: This paper describes the development and evaluation of UNEECON, a framework for jointly predicting deleterious variants and constrained genes. This is certainly an interesting topic in the context of variant effect prediction and interpretation. I find the attempt to unify variant-level and gene-level quite innovative, and it is certainly an approach that will be useful in the study of severe, early onset disorders. Further, the use of a deep neural network to learn parameters relevant to population genetics from millions of variants from gnomAD is a novel contribution to this area. From the perspective of constrained gene prediction, UNEECON results are quite promising. However, I remain skeptical of some of the claims with regards to pathogenicity prediction and the overall argument that this method would be better in practice than those evaluated here (see below). Overall, the paper is clearly written and the methods are outlined satisfactorily (see minor comments for some things that need to be clarified). I outline my comments below:

I thank the reviewer for these comments.

MAJOR:

- A general issue that has plagued the field is the problem of unevenly distributed variant information across genes. Some genes are over-studied and are likely to have more variants identified as pathogenic. More importantly, many genes are likely to contain variants from only one class. UNEECON is interesting in this context that it is trained on genes that are mostly going to contain only benign variants and is evaluated on a ClinVar set that will skew mostly towards pathogenic-only or bi-class genes. Ref. 58 from this paper highlighted a method that performed extremely well in its evaluations when run on “pathogenic-only” or “benign-only” proteins but drastically underperformed on “mixed” genes. Given that UNEECON is heavily influenced by gene-level features, I wonder if it is susceptible to the same issue. One way to test this would be to perform a version of the ClinVar evaluation on only the subset of genes that contain both classes of variants. If performance drops then perhaps unification of variant-level and gene-level information may not be the best approach for variant pathogenicity prediction.

As the reviewer suggested, I compared UNEECON with previously published methods in separating pathogenic missense variants from benign missense variants in the “mixed” genes

which contain both pathogenic and benign variants. Again, UNEECON outperformed the other methods in this setting (Fig. S4).

- On a related note, I am concerned about information leakage between the training set and evaluation sets used in this paper. I agree that UNEECON benefits from actually not using the pathogenic variants in its training and evaluation. However, the sheer size of gnomAD is expected to include every known gene in the training of the deep neural network. Since UNEECON uses gene-level information, there is a distinct possibility that performance may be inflated even after excluding variants in both ClinVar and gnomAD. In fact, Ref. 58 (cited for the circularity and inflation issues) points this out and recommends gene-level partitioning for cross-validation experiments such as those conducted by PolyPhen-2 and MutPred. Of course, in the context of this paper, the proposed experiment would be to train a version of UNEECON without variants in genes from the ClinVar set and evaluate that version on the ClinVar set. That way any gene-level bias in the performance measures would be eliminated.

I agree with the reviewer that supervised machine learning models trained on pathogenic variants could suffer from the inflated performance, because different variants from the same gene may occur both in the training data and in the test data. As mentioned by the reviewer, some genes, such as *TP53* and *BRCA1,* are better studied, so variants in these genes may be overrepresented in both the training and the test data. In this case, supervised machine learning methods can potentially "memorize" which genes have the largest numbers of pathogenic variants in the training data. Because the same set of genes are also overrepresented in the test data, the supervised methods will show an over-optimistic performance due to information leakage.

However, I want to emphasize that UNEECON did not use any known pathogenic variants for training. Instead, UNEECON was trained on gnomAD data, an unbiased map of genetic variants in healthy human populations. The variation of the gene-level density of gnomAD variants reflects the variation of natural selection across genes rather than the ascertainment bias in ClinVar or HGMD. Thus, there is no information leakage between the training and testing data, and UNEECON cannot "cheat" by memorizing the genes overrepresented in ClinVar or HGMD. Therefore, UEECON should not suffer from the same circularity that supervised methods have.

Also, as discussed in the manuscript and in the other reviewer's comments, the key innovation of UNEECON is to combine gene-level constraints and variant-level features using an evolution-guided model. Training a version of UNEECON without gnomAD variants in disease genes will disable UNEECON's ability to learn gene-level constraints in disease genes, leading to an underestimation of UNEECON's performance. In the end, therefore, I have come to the conclusion that it is not appropriate to evaluate UNEECON's performance based on a training set without gnomAD variants in disease genes.

- On page 8, line 254, is it all that surprising that UNEECON-G and pLI scores do not correlate? Intuitively, the impact of missense variants and LOF mutations are going to vary in magnitude

even within the same gene. While a biological explanation (as provided here) for this may very well be plausible, it is more likely that the discrepancies are due to technical reasons. A recent commentary (PMID: 30977936) has touched upon issues related to the methodology and applicability of pLI scores. This commentary highlights the example of BRCA genes that have near-zero pLI scores but are known to harbor several deleterious missense variants.

I agree that the commentary paper (PMID: 30977936) has suggested that gene-level intolerance to LOF mutations may not be a good predictor of pathogenic missense mutations. A key difference between the commentary paper, which only discussed a few genes as examples, and my work is that I carried out a genome-wide investigation of the relationship between missense constraints and LOF constraints. Also, the results in my manuscript indeed strengthened the argument of the commentary paper. Therefore, I have cited the commentary paper and placed my findings in the context of this work.

MINOR:

- The bimodality of the UNEECON score distribution for active sites is worrisome with the peak closer to 0.25 is a little confusing. I interpret this as “there are more variants in active sites that have low UNEECON scores than high.” This is counter-intuitive and warrants some explanation.

I thank the reviewer for this comment. I performed additional analyses to investigate why UNEECON scores showed a bimodal distribution in protein active sites. Because UNEECON scores reflect negative selection on heterozygous missense mutations, I hypothesize that the mode with a lower score corresponds to active sites in recessive genes, and the mode with a higher score corresponds to active sites in dominant genes. In agreement with this explanation, active sites in autosomal recessive disease genes had substantially lower UNEECON scores than those in haploinsufficient and autosomal dominant genes (Fig. S2).

- In the functional analyses related to Fig. 5, are there any interesting depletions? I am curious about the functions of those genes that are tolerant to missense but not to LOF mutations. I am also not sure what “unclassified” means in this context.

The term “unclassified” means the corresponding genes have no known or inferred function in Reactome. I have added a sentence to clarify this point. Also, a fold enrichment below 1 indicates a depletion in the gene set intolerant to both missense and loss-of-function mutations, or equivalently, an enrichment in the gene set tolerant to missense but not to loss-of-function mutations. Thus, the genes that are tolerant to missense but not to loss-of-function mutations are more likely to have no known or inferred function.

- What is the difference between Eqns. 2 and 3? It is difficult to tell with q_i being defined.

Eqn. 2 represents a logistic regression model for parameter estimation, while Eqn. 3 shows the logit of predicted mutability given the estimated parameters. I have modified the Methods section to clarify this point.

- In the Methods section, it would be helpful to readers if a clear account of the parameters to be estimated is provided up front.

I have added sentences to define the parameters to be estimated in the Methods section.

- The paper is missing details of the final model that emerged from the evaluation process, its architecture and its parameters.

I have added the requested details to the Methods section.

- Similarly, the paper lacks details on dataset sizes, particularly in the context of model training and evaluation. How many variants were used to train the deep mixed-effects model? How many variants were included in the evaluations relevant to ClinVar? How many pathogenic and how many benign?

I have added the requested details to the revised manuscript.

- I am also curious about the activation function of the output layer of the neural network. This is of particular relevance to z_{ij} and its scaling relative to u_j. Is there a potential for one quantity to systematically dominate the other in Eqn. 9?

One way to understand the relative contributions of z_{ij} and u_j is to look at Fig. S8. It effectively partitioned the contribution of z_{ij} into the contributions of individual variant features and compared them with the contribution of u_j. This figure showed that u_j had the largest contribution to the output layer of the neural network.

Reviewer #2: The work represents an important advance in prioritization of genes and variants relevant to human disease. it has been known since the introduction of gene level intolerance scoring in 2013 that gene level metrics of the strength of purifying selection provide independent information about variation pathogenicity to the longer established variant level metrics that largely depend on conservation and amino acid substitution features. While attempts have been made previously to integrate both approaches into a single predictive framework these have been based on supervised learning approaches using a set of putatively pathogenic and benign variants. The work here combines a selected set of variant level features with a gene level term and estimates selective constraint operating against all possible gene sequence changes based on human polymorphism data compared against sequence specific mutability. As such, it provides an integrated approach assessing purifying selection operating in the human population.

The authors have rerun the standard assessments used to test both gene level and variant level predictors with generally improved performance both for identifying relevant gene sets (e.g. haploinsufficient genes) and pathogenic variants. In addition to these advances, the model allows some novel biological insights, including explaining an important reasons for discrepancy between intolerance to missense and loss of function variation as being due to the proportion of proteins that is disordered. The model also highlights that the gene level term is more informative than variant level terms which is still not as widely appreciated as it should be. For these reasons the work here represents an important advance in the field.

I thank the reviewer for these comments.

While the paper is generally clearly written and the conclusions generally fair, I do have a couple of relatively minor suggestions for consideration. Perhaps most fundamentally, while the use of UNEECON deep learning model to combine variant features and a gene level term to predict the strength of selection operating against specific alleles is welcome, since it allows non linear combinations of these terms to be learned, it is striking that a linear approximation of the UNEECON model is very highly correlated, suggesting little benefit from the model learning optimum non linear combinations. The authors appropriately use the linear model to infer the relative importance of features, but the very high correlation between the two models suggests the linear modle is likely to have similar performance to UNEECON. Given the more direct interpretability of the linear model, the authors should comment on whether the more complex model is in fact needed for use. The second small point is that some of the comparisons are inappropriate since some of the metrics are used in ways they were not intended for. For example, in Figure 3a representing prediction in distinguishing pathogenic variants gene level metrics such as RVIS are compared directly to UNEECON. As outlined however in the initial work, gene level metrics are intended to be used alongside some version of a variant level predictor (since as emphasized here and in the original publications the two approaches offer independent information). The fair comparison therefore for generating a version of figures 3 focused on variants would be to use a combination of a variant and gene level metric for all those comparisons like RVIS that are gene level metrics. This idea was outlined in the initial publications under the banner of a combined threshold for both gene level and variant level. I have no doubt that UNEECON would still perform better, but one appropriate simple comparison would be to re run these analyses including for example a hard threshold on some appropriate variant score such as PP2 alongside the quantitative gene level score such as RVIS as currently used. Finally, the gene level metrics in use are known to struggle with small genes since there is often not enough polymorphism data to infer selection. The authors should address robustness to gene size.

For the first point, I agree that the additional nonlinearity introduced by neural networks may not be critical for the dataset described in this work. Nevertheless, the deep mixed-effects model has the flexibility of modeling complex interactions between variant-level features, which may be important for analyzing other datasets. I have added sentences to make this point clear.

For the second point, as suggested by the reviewer, I compared UNEECON with the combined-threshold method from the original RVIS paper. As shown in Figure S7, UNEECON outperformed the combined-threshold method in separating pathogenic missense variants from benign missense variants.

For the third point, in the original manuscript, I have already controlled for the impact of gene length on performance evaluation. More specifically, for each disease/essential gene in the positive gene set, I used MatchIt in R to pair it with a negative gene with matched gene length. I have added a few sentences to make this point clear.
