# Supplementary material for: Unified inference of missense variant effects and gene constraints in the human genome
Source: PLoS Genet. 2020 Jul 15;16(7):e1008922. doi: 10.1371/journal.pgen.1008922 (PMC7384676; doi:10.1371/journal.pgen.1008922)
Supplement: Attachment — Submitted filename: Response to the reviewers.pdf [file pgen.1008922.s004.pdf]

Response to Reviewers

I thank the reviewers for their careful reading of my manuscript and their constructive suggestions. My item-by-item responses are interleaved with their comments below (in blue). Note that I used a program, latexdiff, to highlight the changes in my manuscript. Any removed words were crossed out and colored red, whereas added words were colored blue and underlined with a squiggle.

Reviewer #1: The revised version of the paper addresses most of the concerns that I had with the original version of the paper. However, I still remain skeptical of the claim of UNEECON's "unmatched" performance when it comes to pathogenicity prediction. Although the AUCs are indeed higher for UNEECON in Figs. 3, 4, S3 and S4, performances in the most important region of the ROC curves (the low-false-positive-rate region) tend to be on comparable to other methods. I suggest toning down such strong claims made with regards to performance of UNEECON in pathogenicity prediction, when compared to other methods.

I thank the reviewer for this comment. I have removed the word "unmatched" throughout the manuscript and toned down my statements accordingly.

I also would like to follow up on the following statement in the item-by-item response: "Training a version of UNEECON without gnomAD variants in disease genes will disable UNEECON's ability to learn gene-level constraints in disease genes, leading to an underestimation of UNEECON's performance." This gets to the actual motivation behind my comment. If gene-level constraints are that important to UNEECON's performance, then it is expected that UNEECON will underperform when attempting to predict a pathogenic variant in a gene with no previous known disease association. The gnomAD subset that does not overlap with ClinVar serves as a proxy for such genes as it is quite comprehensive in the coverage of the genome. My original concern was that UNEECON may simply be good at separating disease-associated genes (which is as the author correctly said is subject to ascertainment bias) from those in gnomAD, and that this was a major driver of variant-level predictive performance. This is somewhat alleviated through the inclusion of Fig. S4 but a true test of UNEECON's ability to contribute to novel discoveries is in its ability to make correct variant-level predictions in "undiscovered" disease genes. If an experiment to test this seems infeasible, it would be helpful to clearly state this as a limitation of the model in the Discussion section.

I thank the reviewer for this idea. As suggested, I retrained the UNEECON model on a dataset without known disease genes. Then, I evaluated the performance of this version of UNEECON in separating pathogenic missense variants from benign missense variants in disease-causing genes. Because disease-causing genes were not used in the training step, UNEECON effectively substituted the gene-level random-effect term with its genome-wide average in the prediction step, forcing UNEECON to make predictions solely based on variant-level features. Again, UNEECON outperformed alternative methods in this setting (Fig S4B). Therefore,

UNEECON can still accurately predict pathogenic variants when the information of gene-level constraints is absent.

Reviewer #2: the authors have done a thorough job of responding to the reviews and I have no further comments.

I appreciate the reviewer’s help in improving this manuscript.
